# Ensemble Classification of Alzheimer's Disease and Mild Cognitive Impairment Based on Complex Graph Measures from Diffusion Tensor Images

**DOI:** 10.3389/fnins.2017.00056

**Published:** 2017-02-28

**Authors:** Ashkan Ebadi, Josué L. Dalboni da Rocha, Dushyanth B. Nagaraju, Fernanda Tovar-Moll, Ivanei Bramati, Gabriel Coutinho, Ranganatha Sitaram, Parisa Rashidi

**Affiliations:** ^1^Department of Biomedical Engineering, University of FloridaGainesville, FL, USA; ^2^Brain and Language Lab, Department of Clinical Neuroscience, University of GenevaGeneva, Switzerland; ^3^Department of Computer and Information Science and Engineering, University of FloridaGainesville, FL, USA; ^4^D'Or Institute for Research and Education (IDOR)Rio de Janeiro, Brazil; ^5^Institute for Biomedical Sciences, Federal University of Rio de JaneiroRio de Janeiro, Brazil; ^6^Institute for Biological and Medical Engineering, Schools of Engineering, Biology and Medicine, and Department of Psychiatry and Section of Neuroscience, Pontificia Universidad Católica de ChileSantiago, Chile; ^7^Laboratory for Brain-Machine Interfaces and Neuromodulation, Pontificia Universidad Católica de ChileSantiago, Chile

**Keywords:** Alzheimer's disease, ensemble classification, diffusion tensor images, machine learning, graph measures

## Abstract

The human brain is a complex network of interacting regions. The gray matter regions of brain are interconnected by white matter tracts, together forming one integrative complex network. In this article, we report our investigation about the potential of applying brain connectivity patterns as an aid in diagnosing Alzheimer's disease and Mild Cognitive Impairment (MCI). We performed pattern analysis of graph theoretical measures derived from Diffusion Tensor Imaging (DTI) data representing structural brain networks of 45 subjects, consisting of 15 patients of Alzheimer's disease (AD), 15 patients of MCI, and 15 healthy subjects (CT). We considered pair-wise class combinations of subjects, defining three separate classification tasks, i.e., AD-CT, AD-MCI, and CT-MCI, and used an ensemble classification module to perform the classification tasks. Our ensemble framework with feature selection shows a promising performance with classification accuracy of 83.3% for AD vs. MCI, 80% for AD vs. CT, and 70% for MCI vs. CT. Moreover, our findings suggest that AD can be related to graph measures abnormalities at Brodmann areas in the sensorimotor cortex and piriform cortex. In this way, node redundancy coefficient and load centrality in the primary motor cortex were recognized as good indicators of AD in contrast to MCI. In general, load centrality, betweenness centrality, and closeness centrality were found to be the most relevant network measures, as they were the top identified features at different nodes. The present study can be regarded as a “proof of concept” about a procedure for the classification of MRI markers between AD dementia, MCI, and normal old individuals, due to the small and not well-defined groups of AD and MCI patients. Future studies with larger samples of subjects and more sophisticated patient exclusion criteria are necessary toward the development of a more precise technique for clinical diagnosis.

## Introduction

Alzheimer's disease (AD), a neurodegenerative disorder characterized by progressive dementia, is the seventh leading cause of death in the United States (Heron and National Center for Health Statistics, 2009). AD is the most common form of dementia and currently affects over five million Americans. This number will grow to as many as 14 million by year 2050 (Brookmeyer et al., 2007). AD may affect people in different ways, but the most common first symptom is the inability to remember new information (Burns and Iliffe, 2009). The progression of AD toward other brain regions causes disruption of the daily life, changes in personality, and withdrawal from work and social activities. In advanced stages, the individual is unable to communicate with others and to recognize close relatives. Finally, difficulty executing motor tasks precedes death (Alzheimer's Association, 2011). There is currently no cure for AD, while most drugs only alleviate the symptoms (Jack et al., 2008).

The transitional stage in which the patient is not considered normal, but does not meet the criteria for dementia is called Mild Cognitive Impairment (MCI). MCI consists of heterogeneous symptomatology, and includes prodromal AD stages as well as mild stages of other types of dementing disorders (Dubois and Albert, 2004). Promodal AD brains have partial similarities to more severe AD brains, while mild stages of other types of dementing disorders have different features by which it would be possible to distinguish non-AD MCI patients from the AD patients.

According to the International Working Group-2 (IWG-2) criteria by Dubois et al. (2014), the diagnostic biomarkers for AD are the clinical phenotype (typical or atypical presentations of AD related neuropsychological deficits), as well as the pathophysiologic presence of extracellular amyloid plaques and intra-neuronal neurofibrillary tangles, as defined by Alzheimer (1907), which are associated with synaptic loss, functional neurotransmission deficits, and neuronal death. The pathophysiologic markers of AD can be accessed by positron emission tomography (PET; Drzezga et al., 2003) and cerebrospinal fluid tests (Tapiola et al., 2009). Topographic biomarkers such as F-2-fluoro-2-deoxy-D-glucose (PET-FDG; Bohnen et al., 2014) and Magnetic Resonance Imaging (MRI) hippocampal volumetry (Sarazin et al., 2010) are proposed to track disease progression.

The diagnosis of MCI due to AD is possible nowadays (Alzheimer's Association, 2011; Dubois et al., 2014), based on cognitive impairments with mild impact on the daily activities, and considering some excluding clinical criteria. The diagnosis is also possible based on positive physio-pathologic markers of AD such as an abnormal level of amyloid beta and/or tau in the cerebrospinal fluid (CSF), or an abnormal load of amyloid beta and/or tau in the brain as revealed by PET. Parallel early evidences of AD are reduction of brain metabolism in the parietal, temporal and hippocampal regions measured by FDG-PET, and hippocampal atrophy revealed by structural MRI.

In recent years, structural MRI experiments (e.g., Wolz et al., 2011; Casanova et al., 2012; Lillemark et al., 2014) have revealed prospective biomarkers with top achievements reaching values superior to 80%. However, it is not yet clear if structural MRI is able to detect earlier biomarkers of AD compared to other MRI modalities, such as functional and diffusion MRI. Several studies have investigated the use of resting-state functional Magnetic Resonance Imaging (rs-fMRI), a non-invasive method for automatic diagnosis of brain diseases (e.g., Chen et al., 2011; Brier et al., 2012; Tang et al., 2013; Hoekzema et al., 2014; Zeng et al., 2014). Chen et al. (2011) achieved accuracy slightly higher than 80%. In that approach, Pearson's correlation coefficient (r) of pairwise regions of interests (ROIs) was used for distinguishing AD patients from healthy subjects in a group analysis. However, given that the correlation coefficient represents only a linear relationship, it follows that if the underlying ground truth is not linear, the result of such an analysis will be inaccurate. Furthermore, the above approach cannot be applied to diagnosing individual patients in a clinical setting as the correlation analysis is calculated on group data. Hence, to study the complexities of brain networks and to identify brain disorders (e.g., AD) only by studying the rs-fMRI correlation between different brain regions may not be sufficient. A more advanced approach is necessary to study the complexities of brain networks, and the diagnosis of single subject data.

Complex networks can be analyzed efficiently using graph theory. Graph theory models each brain region as a node and the relationship between two regions as an edge. Recent studies have focused on the use of graph theoretical measures on the structural brain networks (Bassett et al., 2008; Bassett and Bullmore, 2009; Bullmore and Sporns, 2009; Lynall et al., 2010; Wang et al., 2010; Várkuti et al., 2011; Zhang et al., 2011; Khazaee et al., 2015). Depending on the type of data, various graphs can be constructed. For example, representations of neural networks can be constructed using microscopic data (e.g., Chatterjee and Sinha, 2007), where nodes represent neurons and edges axons connecting neurons to each other. Recent studies have shown that graph theoretical measures are vital in identifying network measures of psychiatric and neurological diseases. Graph theory not only can be used to study various network properties such as small-world property or efficiency of the information transfer, but it can also be employed in medical applications and disease diagnosis. For example, Bassett et al. (2008) used graph theoretic methods to show that patients suffering from Alzheimer's disease and schizophrenia have abnormal network configurations.

Recently, machine learning has been used in the detection of diseases by recognizing physiological patterns (biomarkers) of healthy and pathological conditions (Teipel et al., 2008; Mapstone et al., 2014). There has been a growing interest in applying machine learning techniques on DTI data of Alzheimer's patients (Hahn et al., 2013). Recent studies have derived connection matrices as well as graph metrics from DTI data (Lo et al., 2010) of Alzheimer's patients. Dyrba et al. (2013) report a machine learning approach for discriminating between Alzheimer's disease and healthy controls using fractional anisotropy (FA) values as input features, achieving 80% of accuracy. However, these studies have used information such as Pearson's product moment correlation coefficient between brain regions (Wang et al., 2006; Chen et al., 2011), regional homogeneity (ReHo), and amplitude of low-frequency fluctuations (ALFF; Dai et al., 2012; Zhang et al., 2012), and a limited number of network measures as discriminant features (Li et al., 2013). Additionally, these studies considered only global network measures but ignored local network measures. Local network measures that are indicators of individual network elements (such as nodes or links) typically quantify connectivity profiles associated with these elements and reflect the way in which these elements are embedded in the network. Hence, one major focus of the current research is to identify more effective local and global structural properties of the brain network to enhance the performance of pattern classification.

The purpose of current study is to implement a system that efficiently classifies AD patients from MCI patients and healthy subjects (CT). We analyzed DTI data of 15 AD patients, 15 MCI patients, and 15 healthy volunteers, and performed pattern classification of the three categories of subjects based on graph measures. We considered pair-wise class combinations of subjects and defined three separate classification tasks, i.e., AD-CT, AD-MCI, and CT-MCI. An ensemble classification module was used to perform the classification tasks. A limitation of this study is the small sample of amnesic MCI, as those could be a mix of people with AD and other dementing disorders such as dementia with Lewy body dementia. Due to the limited sample size, this study can be regarded as a “proof of concept.”

## Materials and methods

The study was performed in three stages: data collection and processing, feature extraction, and classification. Data acquisition was performed in an MR scanner, and data processing included a preprocessing phase containing realignment, coregistration, normalization, and segmentation of the data. Fractional anisotropy based tractography, and feature extraction was performed based on graph theory by calculating local network centrality measures (Freeman, 1977). Pattern classification was performed by the method of ensemble classification (Rokach, 2010).

### Data collection and processing

#### Subjects and data acquisition

Forty-five adults consisting of 15 AD patients, 15 MCI patients, and 15 healthy volunteers were recruited for DTI data acquisition. DTI scans were acquired in the Institute D'or (Rio de Janeiro, Brazil) on a Philips Achieva 3.0 Tesla MR scanner, using a spin echo (SE) sequence with the following parameters: repetition time (TR) = 5620 ms, echo time (TE) = 65 ms, flip angle = 90°, acquisition matrix = 96 × 96 with a spatial resolution of 2.5 × 2.5 mm, 60 transversal slices with thickness = 2.5 mm, 32 gradient directions, and *b*-value 1000 s/mm^2^.

The adults in study were referred for neuropsychological evaluation by their physicians, because of memory complaints to discriminate among normal aging, MCI, or dementia. Participants with tumor, stroke, traumatic brain injury, or hydrocephalus were excluded from the experiment. AD and MCI diagnoses were made by consensus among a trained, a neuropsychologist and a psychiatrist, based on DSM-IV criteria, MRI overview, clinical data and neuropsychological tests. The AD patients had mild dementing disorders and the supportive tests for first AD diagnosis were performed in parallel to the MR/DTI acquisition. AD diagnoses were performed considering NINCDS-ADRDA criteria (Knopman et al., 2001). All individuals underwent a comprehensive evaluation for diagnostic propose, including the following tests, whose quantitative results are available in the Appendix (Table A2) in Supplementary Material:

1. Mini Mental State Evaluation (MMSE),
2. Span (digit and spatial) forward and backward,
3. Clock Drawing Test (CDT),
4. Verbal Fluency (semantic and letter),
5. Family Pictures,
6. Geriatric Depression Scale (Yesavage et al., 1983),
7. Memory Assessment Complaints Questionnaire (Crook et al., 1992).

The MCI patients were all from the amnestic category. We did not apply the Hachinski Ischemic Scale. We used Petersen criteria (Petersen et al., 1997, 2001) for MCI diagnosis, including:

1. Memory problems,
2. Objective memory disorder,
3. Absence of other cognitive disorders or repercussions on daily life,
4. Normal general cognitive function,
5. Absence of dementia.

Healthy volunteers were selected by matching age and education level to the MCI patients and AD patients (Table 1). A two-tailed independent *t*-test was used to determine whether the differences in age and educational level between the groups were statistically significant. The results show *P*-values between 0.9 and 1.0 (Table 2), which confirms no significant difference (Panagiotakos, 2008) between the groups. The connectivity regions of participants' DTI data were defined in terms of Brodmann areas. A Brodmann area is a region of the cerebral cortex in the human brain, defined by cytoarchitectonic and histological analysis of the structure and organization of cells (Brodmann, 1909).

**Table 1:** **The 45 participants of the study and their corresponding class, sex, age and educational level**.

**The 15 control volunteers**				**The 15 Mild Cognitive impairment patients**				**The 15 Alzheimer's disease patients**			
**Subject ID**	**Age (years)**	**Education (years)**	**Sex**	**Subject ID**	**Age (years)**	**Education (years)**	**Sex**	**Subject ID**	**Age (years)**	**Education (years)**	**Sex**
01	85	11	Female	16	80	08	Female	31	74	08	Female
02	81	08	Female	17	81	04	Female	32	73	15	Female
03	76	17	Female	18	68	15	Female	33	77	04	Female
04	74	11	Female	19	73	15	Male	34	68	15	Female
05	74	04	Female	20	88	08	Male	35	68	15	Male
06	78	11	Female	21	84	11	Female	36	71	11	Female
07	74	15	Male	22	70	20	Male	37	74	20	Male
08	83	15	Male	23	67	04	Male	38	84	11	Male
09	62	11	Female	24	71	11	Female	39	75	11	Male
10	67	11	Female	25	71	20	Female	40	86	15	Female
11	77	08	Female	26	80	14	Female	41	73	11	Female
12	78	11	Female	27	77	08	Female	42	80	15	Male
13	72	11	Female	28	70	15	Male	43	61	04	Male
14	61	21	Male	29	66	15	Female	44	72	15	Female
15	77	15	Male	30	69	11	Female	45	81	11	Female
Mean	74.6	12.0	73%F	Mean	74.3	11.9	67% F	Mean	74.5	12.1	60% F
			27% M				33%M				40%M
Standard deviation	6.9	4.1	–	Standard deviation	6.8	5.0	–	Standard deviation	6.5	4.3	–

**Table 2:** *****P***-values by age and educational level on binary discrimination**.

**Binary discrimination**	**Age(***p***-value)**	**Educational level(***p***-value)**
Control vs. MCI	0.916	1.000
Control vs. Alzheimer	0.957	0.931
MCI vs. Alzheimer	0.957	0.938

For each subject, T1-weighted structural images and DTI images were acquired by a gradient recalled echo (GRE) scanning sequence, with the following parameters: TR = 7.16 ms, TE = 3.41 ms, flip angle = 8°, acquisition matrix = 480 × 480 with spatial resolution 0.5 × 0.5 mm, and 340 sagittal slices with thickness of 0.5 mm.

#### Data processing

**Figure 1:**
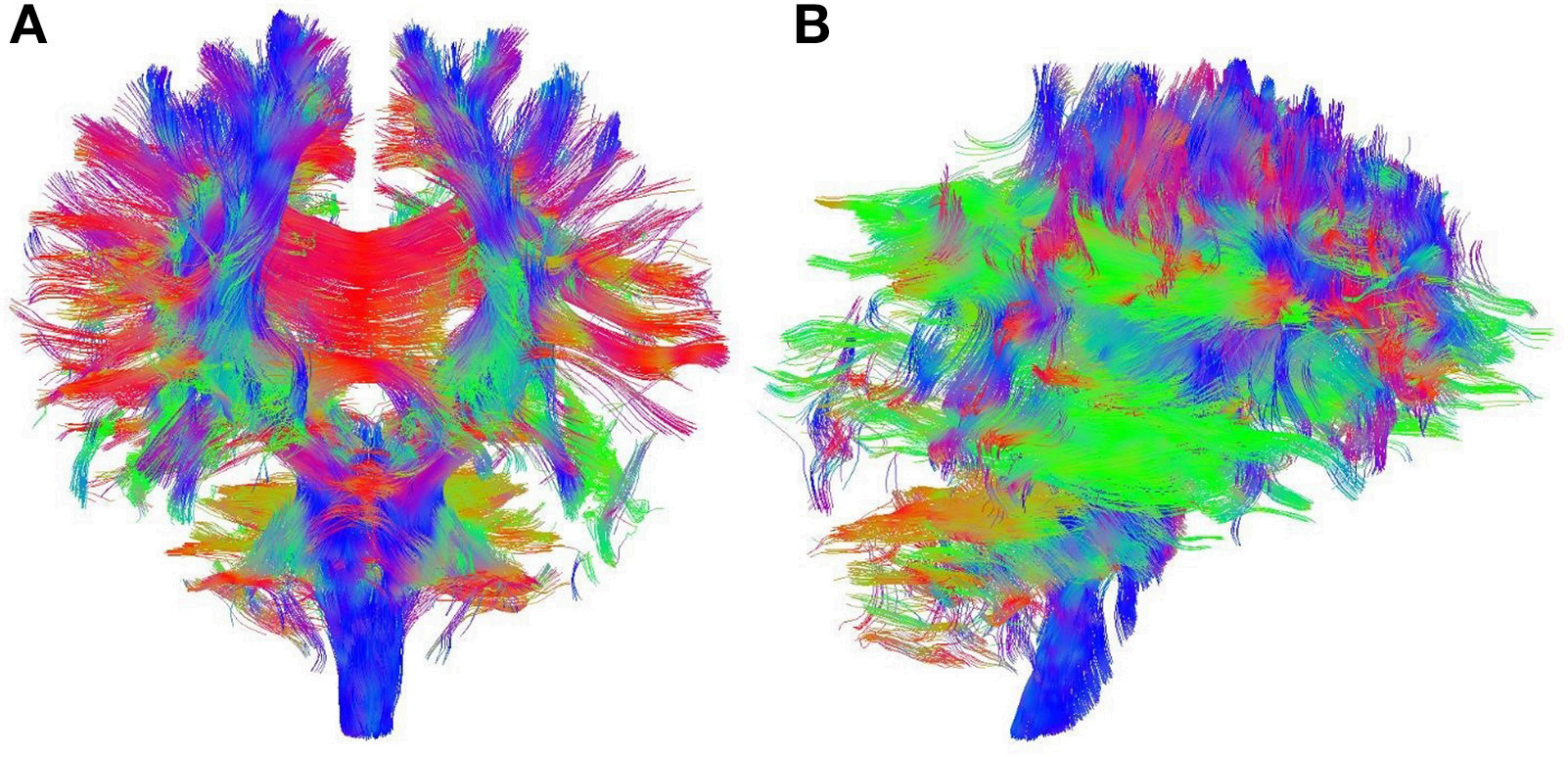
**Sample output of whole brain tractography of DTI data**. Colored lines represent estimates of brain's structural connectivity. Blue lines show connections in superior-inferior direction, green lines show anterior-posterior direction, and red lines represent medial-lateral direction. **(A)** Coronal view. **(B)** Sagittal view.

**Figure 2:**
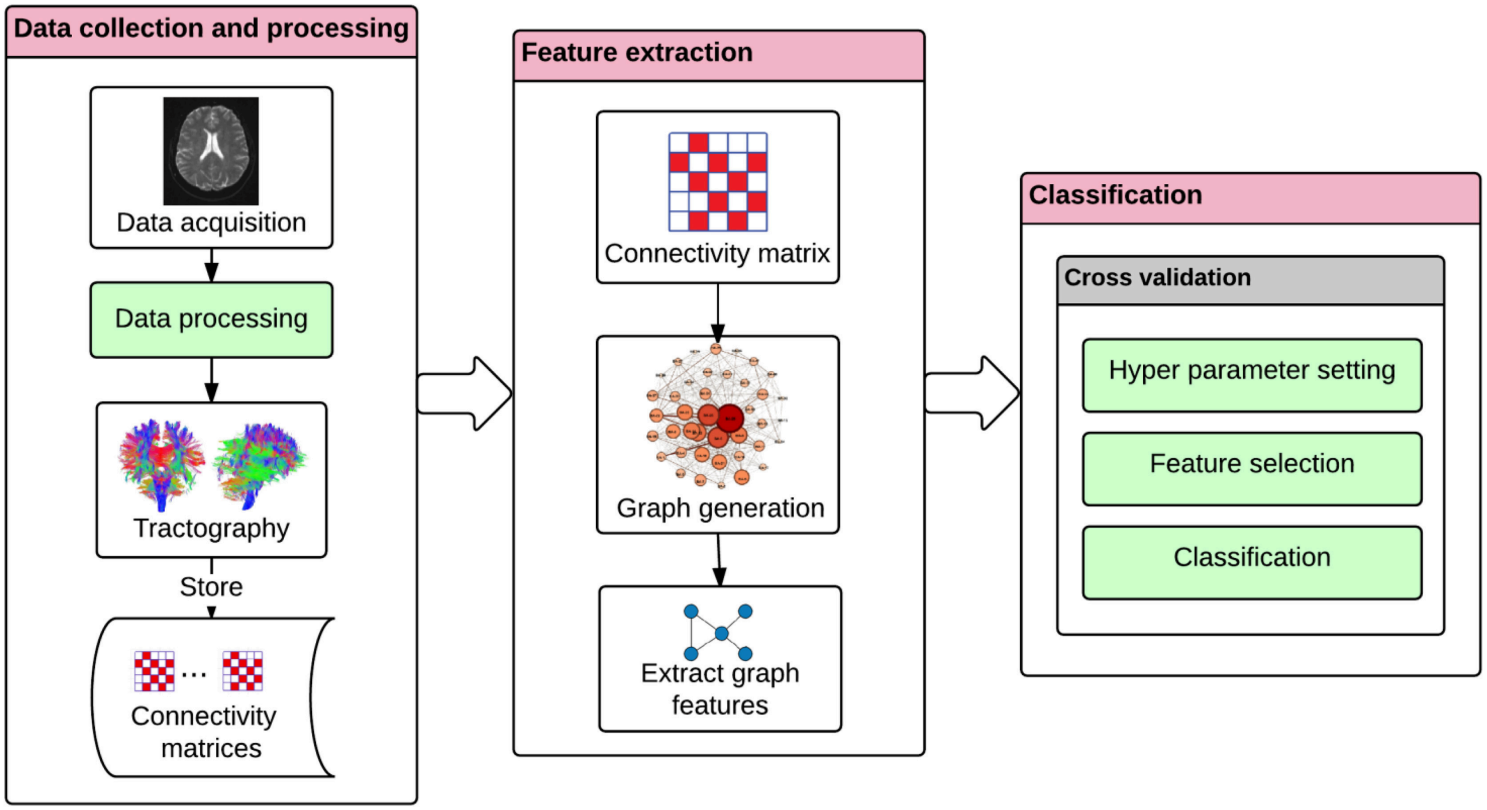
**The classification approach for diagnosing Alzheimer's disease**. The DTI tractography provides information on brain's connectivity fibers. The connectivity matrix is extracted by the discretization of the brain connection fibers, and by representing Brodmann areas as nodes and the white matter bundles interconnecting different gray matter areas as the edges. The interpretation of the connectivity matrix as a graph allows the extraction of the graph features. These graph features can then be used in a machine learning framework. The machine learning module includes the following procedures: hyper-parameter setting, feature selection, and classification based on a nested cross-validation procedure.

The DTI data was preprocessed with realignment, coregistration, and normalization using the software, SPM8 (Friston, 1996), and segmentation by using the software, DSI Studio (Yeh and Tseng, 2011). FA was calculated for each voxel of each subject's brain volume, generating a FA map for each subject. FA is commonly considered to be an indicator of structural brain connectivity, since it is sensitive to the axonal structure (Basser and Pierpaoli, 1996). Important factors influencing FA is the integrity of axons and their myelin sheaths (Damoiseaux et al., 2009). Subsequently, tractography (Le Bihan and Breton, 1985), a 3D modeling technique that visually represents neural tracts using DTI data, was performed to obtain the Connectome (Sporns et al., 2005). Tractography (Figure 1) was performed deterministically by using the entire brain as the seed with the toolbox, DSI Studio (Kreher et al., 2008; Yeh et al., 2013). This process was performed according to the following parameters: fractional anisotropy (FA) threshold = 0.1, number of seed points = 1,000,000, maximum angle = 60°, step size = 1.25 mm, length constraint = 25–100 mm, and no spatial smoothing^1^. Connectivity matrices were obtained and stored accordingly. We considered 41 Brodmann areas according to the coordinates established in DSI studio (Yeh et al., 2013). In a connectivity matrix, rows and columns of the matrix represent different Brodmann areas. Each cell of the matrix represents a distinct connection between two Brodmann areas corresponding to specific row and column. Figure 2 shows the different stages of our approach. Each block in the figure represents a stage in the classification procedure.

### Feature extraction

#### Graph generation and construction of structural brain networks

**Figure 3:**
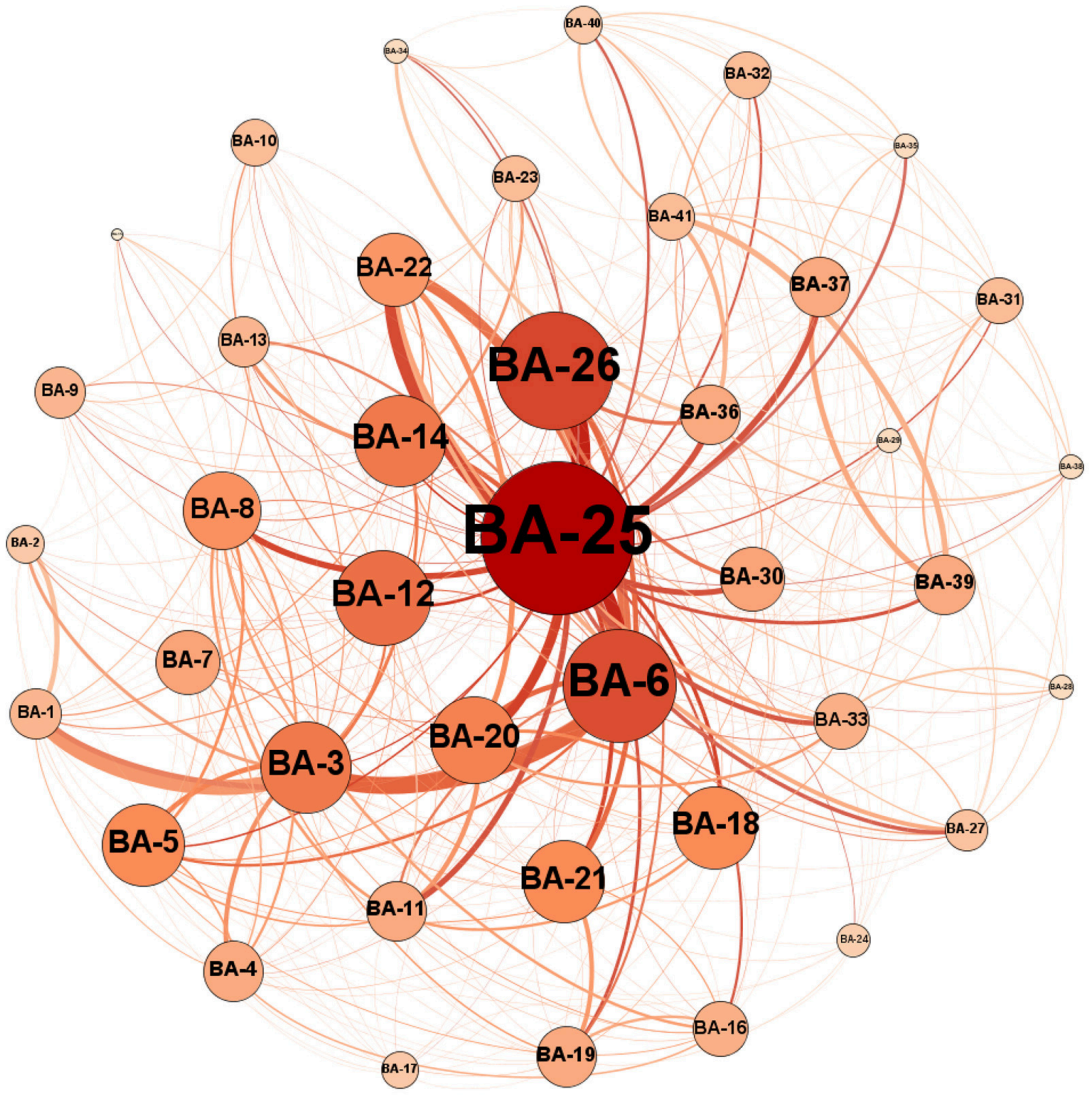
**An example of a graph for an MCI patient where each circle represents one node, i.e., a Brodmann area (BA) in the brain, and connections between nodes represent edges, i.e., white matter connections**. The numbers on the nodes represent the label of the Brodmann areas. The diameter of each node is proportional to the number of connections of its corresponding Brodmann areas, and the thickness of each line represents the weight or connection strength of the edge.

In graph theory, a graph is defined as a set of nodes and edges (Bullmore and Sporns, 2009). Using connectivity matrices obtained above, we generated the corresponding graphs where the nodes are brain regions and edges are the bundles of nerves connecting those regions (Gong et al., 2009). In this manner, a graph was generated for each subject using the connectivity matrices generated in the previous data processing. The graphs we constructed were all undirected, i.e., the nodes are connected together by bidirectional edges. Brodmann areas were represented as network nodes. Based on the 41 Brodmann areas considered in this analysis, each network was composed by a total of 41 nodes. An edge was marked between two nodes, representing two Brodmann areas, if there was at least one fiber connecting them. Otherwise these two nodes were not considered directly connected, although they could still be connected via another node or nodes. We used the weight (W_ij_) value of the edge to describe the strength of the connectivity of Brodmann areas *i* and *j*. We constructed the connectivity matrix as a weighted matrix for each subject. As there were 41 Brodmann areas, the maximum number of all possible edges was 820 [*N* (*N* − 1)/2; *N* = 41]. The number of nodes (41) was the same for each subject since each brain was parcellated using the same scheme. However, subjects differed in the structure of their graph in terms of both the number and weighting on the edges. The numbers of edges were likely to differ for different graphs as the result of varying strengths and presence/absence of the connectivity of Brodmann areas in the subjects. Figure 3 shows the graph of an MCI patient as an example.

#### Extracting graph features

**Figure 4:**
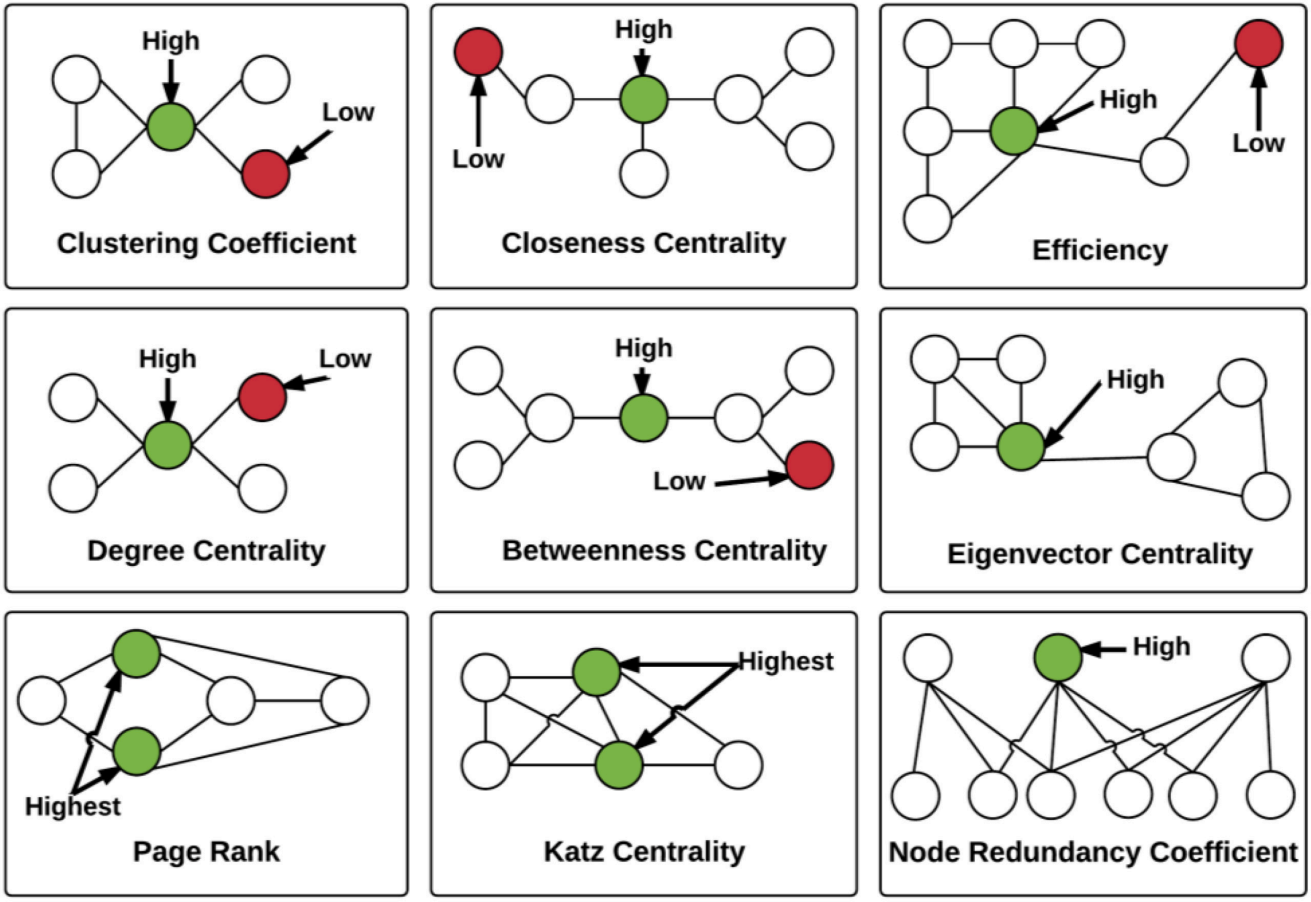
**Illustration of the local graph measures**. For each graph measure, a node with high measure value and a node with low measure value are identified. Note that Load and HITS centralities are not illustrated. The measures are described in detail in the Section Extracting Graph Features.

The network properties that we computed included the following: closeness centrality, betweenness centrality, eigenvector centrality, Katz centrality, hyperlink-induced topic search (HITS) centrality, degree centrality, clustering coefficient centrality, load centrality, and efficiency and node redundancy coefficient (Figure 4). The definition of the different network measures are presented below.

#### Closeness centrality

Closeness centrality indicates how close a node is to all the other nodes in a network (Wasserman and Faust, 1994). The centrality of a given node is the sum of the geodesic distances (shortest paths from one node to other nodes). Closeness centrality describes the extent of influence of a node on the network. Closeness can be regarded as a measure of how long it will take to spread information from node *i* to all other nodes sequentially. Consider a connected weighted undirected graph *G* = (*V, E*) with *n* vertices and *m* edges (|*V*| = *n*, |*E*| = *m*). Let *d(v, u)* denote the length of the shortest path between v and u. The closeness centrality C_v_ (Sabidussi, 1966) of node *v* is defined in Equation (1):

(1) $$C_{\text{v =}}\;\frac{n-1}{\sum_{u\in \;V}d(v,u)}$$

##### Betweenness centrality

Betweenness centrality is a measure of the number of times a node is seen to lie along the shortest path between two other nodes (Brandes, 2001). It is equal to the number of shortest paths from all vertices to all others that pass through that node. A node with high betweenness centrality has a large influence on the transfer of items through the network, under the assumption that item transfer follows the shortest paths. Studies have illustrated that the brain uses the shortest path during information processing to save time and energy (Klyachko and Stevens, 2003). Hence, we selected closeness and betweenness centrality as measures reflecting the shortest paths in our model. For a graph *G* = (*V, E*), betweenness centrality C_B_(v) (Brandes, 2001) is given in Equation (2).

(2) $$C_{\text{B}}(\text{v})=\sum_{s,t:s\neq t\neq v}\frac{\sigma_{v}(s,t)}{\sigma (s,t)}$$

Here σ(*s, t*) is the number of shortest paths from *s* to *t*, and σ_v_(*s, t*) is the number of shortest paths from *s* to *t* that pass through *v*.

##### Eigenvector centrality

Eigenvector centrality assigns relative scores to all nodes in the network based on the concept that connections to high-scoring nodes contribute more to the score of the node in question than connections to low-scoring nodes. Eigenvector centrality is calculated by assessing how well-connected an individual is to the parts of the network with the greatest connectivity (Gould, 1967). Recent studies have shown that eigenvector centrality identifies the most prominent regions in a network (Binnewijzend et al., 2014). For a weighted, undirected graph *G* = (*V, E*) with *n* vertices and *m* edges, connectivity matrix *A*, Eigen value λ and corresponding eigenvector × satisfying the condition *Ax* = λ*x*, the eigenvector centrality C_e_(v) (Newman, 2008) of node *v* is given in Equation (3):

(3) $$C_{\text{e}}(\text{v})=\frac{1}{\lambda (max)}\sum_{i\;=\;1}^{n}A\left(v,i\right)x_{\text{i}}$$

The above equation represents the *v*^th^ entry in the eigenvector × corresponding to the largest eigenvalue λ (max).

##### Katz centrality

Katz centrality measures the degree of influence of a node in a network. Unlike typical centrality measures which consider only the shortest path (the geodesic) between a pair of actors, Katz centrality measures influence by taking into account the total number of walks between a pair of nodes. Katz centrality computes the relative influence of a node within a network by measuring the number of the immediate neighbors (first degree nodes) and also all other nodes in the network that connect to the node under consideration through these immediate neighbors (Katz, 1953). It is closely related to eigenvector centrality. For a connected, weighted and undirected graph *G* = (*V, E*) with *n* vertices, *m* edges and connectivity matrix *A*, Katz centrality C_k_(v) (Junker and Schreiber, 2011) of node v is given in Equation (4).

(4) $$C_{\text{k}}(\text{v})=\sum_{k\;=\;1}^{\infty }\sum_{u\;=\;1}^{n}\alpha^{k}(A^{k}{)}_{\text{uv}}$$

In Equation (4), α, the attenuation factor, is a value which is chosen to be smaller than the reciprocal of the absolute value of the largest eigenvalue of adjacency matrix *A*. The powers of *A* indicate the presence or absence of edges between two nodes through intermediaries. For instance in matrix *A*^4^ if the element (*a*_1_, *a*_6_) = 1, indicates that through some first and second degree neighbors of node 1, nodes 1, and 6 are connected.

##### HITS centrality

HITS centrality measures the hub and authority centrality scores of a valued network. Hubs and authorities are a natural generalization of eigenvector centrality. There are two scores for each node in a network, a hub and an authority score. A high hub actor points to many good authorities and a high authority actor receives from many good hubs. The authority score of a vertex is therefore proportional to the sum of the hub scores of the vertices on the in-coming ties and the hub score is proportional to the authority scores of the vertices on the out-going ties. Theoretically, consider a connected, weighted and undirected graph *G* = (*V, E*) with *n* vertices and *m* edges (|*V*| = *n*, |*E*| = *m*). The HITS algorithm (Von Ahn, 2008) consists of a series of iterations with the following steps:

1. Authority update rule: For a given node *v*, the authority update score is the summation of hub scores of each node which point to node and is denoted in Equation (5):
  (5) $$\text{auth }(\text{v})=\sum_{i\;=\;1}^{n}\;hub(i)$$
2. Hub update rule: For a given node *v*, update of its hub score is the summation of authority scores of all nodes pointing to *v* which is denoted in Equation (6):
  (6) $$\text{hub }(\text{v})=\sum_{i\;=\;1}^{n}\;auth(i)$$

##### Degree centrality

Degree (also called valency) of a node in undirected graphs is the total amount of edges directly connecting that node to any other node. It is given by the sum of numbers of edges connecting the nodes. A node with a higher degree of connectivity may be more relevant to the network in terms of number of connections, but it does not measure the importance of each of those connections. Degree has its neurobiological interpretation: a region with high degree interacts structurally and functionally with many other regions in a brain network (Sporns and Kötter, 2004). For a connected weighted undirected graph *G* = (*V, E*) with *n* vertices and *m* edges (|*V*| = *n*, |*E*| = *m*), the degree centrality C_D_(v) (Freeman, 1979) of node *v* is defined in Equation (7).

(7) $$C_{\text{D}}(\text{v})=\frac{\text{deg}\;(\text{v})}{n-1}\;$$

In Equation (7), deg (v) is the number of edges incident upon a node.

##### Clustering coefficient

The Clustering coefficient of a node in an undirected graph is a measure that quantifies the fraction of direct connections between the nearest neighbor nodes that exist out of all possible direct connections amongst those nearest neighbor nodes (Watts and Strogatz, 1998). It quantifies the presence of clusters or groups within a network as a measure of functional segregation in the brain, and denotes ability for specialized processing to occur within densely interconnected groups of brain regions. Therefore, it was also added to our model. For a connected, weighted and undirected graph *G* = (*V, E*) with *n* vertices and *m* edges (|*V*| = *n*, |*E*| = *m*), the clustering coefficient centrality C_v_ (Watts and Strogatz, 1998) of node *v* is given in Equation (8).

(8) $$C_{\text{v}}\;=\;\frac{2e}{k(k-1)}$$

In Equation (8), *k* is the number of neighbors of *v*, and *e* is the number of connected pairs between all neighbors of *v*. Clustering coefficient is a ratio *N*/*M*, where *N* is the number of edges between the neighbors of *v*, and *M* is the maximum number of edges that could possibly exist between the neighbors of *v*.

##### Efficiency

The Efficiency of a node in a network is a measure of how efficiently, in terms of path length, information can be exchanged with other nodes. Thus, the efficiency is inversely related to the shortest path length between the nodes and is used to evaluate how easily a node can be reached from other nodes. Therefore, the efficiency of a node is the inverse of the harmonic mean of the distances to other nodes (Latora and Marchiori, 2001).

##### Page rank

Page rank is a well-known algorithm, originally used by Google to rank websites for their search engine (Sullivan, 2007). Page rank is another proxy for evaluating the importance of a node in a network, by assigning a probability of visiting that node after many steps and extent of time. Theoretically, first the standard adjacency matrix is normalized such that the columns of the matrix sum to 1. Next, page rank values are obtained as the values in the eigenvector with the highest corresponding eigenvalue of the normalized adjacency matrix (Page et al., 1999). In our case, page rank can highlight the Brodmann areas with higher number of external links, and the ones that are more frequently addressed. That is, if a Brodmann area is receiving more links from other areas, it might have more important role within the brain.

##### Load centrality

The load centrality of node *i* in a network is calculated based on the fraction of all the shortest paths that pass through the node *i* (Goh et al., 2001). Load centrality is different from betweenness centrality, although they are both related to the shortest path (Brandes, 2008). Theoretically, load centrality is defined based on a hypothetical flow process. It is assumed that each node in a network sends a unit amount of a given commodity to every other node in the network, regardless of any edge or node capacity limit. Using a priority system, the commodity is passed to the neighbors of the node that are closest to the target destination. If there is a tie, i.e., more than one node candidate, the commodity is divided equally among them. The load of a node is calculated as the total amount of commodity passing through it, within the whole process (Goh et al., 2001). Load centrality is a potential alternative to betweenness centrality which provides a complementary view over the flow structures in the network.

##### Node redundancy coefficient

The redundancy coefficient of node *i* is calculated based on the fraction of pairs of neighbors of node *i* that are also connected to other nodes in the network. This measure captures the nodes that are of lower importance and can be induced by other nodes in the network. For any node *i*, the node redundancy coefficient of node *i, RC*_*i*_, is defined as in Equation (9).

(9) $$RC_{i}=\frac{\left|\left\{\left\{j,k\right\}\subseteq N\left(i\right),\;\;∋i^{\prime }\neq i,\left(i^{\prime },j\right)\in E\;and\;(i^{\prime },k)\in E\right\}\right|}{\frac{\left|N(i)\right|\left(\left|N(i)\right|\;-\;1\right)}{2}}$$

In Equation (9), *N(i)* is the set of neighbor nodes for node *i*, and *E* is the set of edges of the network (Latapy et al., 2008). The redundancy coefficient ranges from 0 to 1, where larger numbers for a node mean higher redundancy. Despite the differences in definitions, node redundancy coefficient can be considered as a generalization of clustering coefficient to squares, i.e., *C*_4_, introduced by Lind et al. (2005), which calculates the probability of a situation in which a node in the network has two neighbors where these two neighbors have another neighbor in common. If a network is fully connected (where all nodes are connected to all other nodes), redundancy coefficient will be extremely high, since a damage in removal of a node does not affect the network, due to the existence of parallel pathways. In case of brain networks, it could be useful to evaluate the damage caused by illness known to affect gray matter connections such as Schizophrenia.

The above mentioned 11 network properties were computed for each node (i.e., 41 Brodmann areas) in the graph to form a vector of 451 features. Feature selection was then performed on the feature vector.

### Classification model

**Figure 5:**
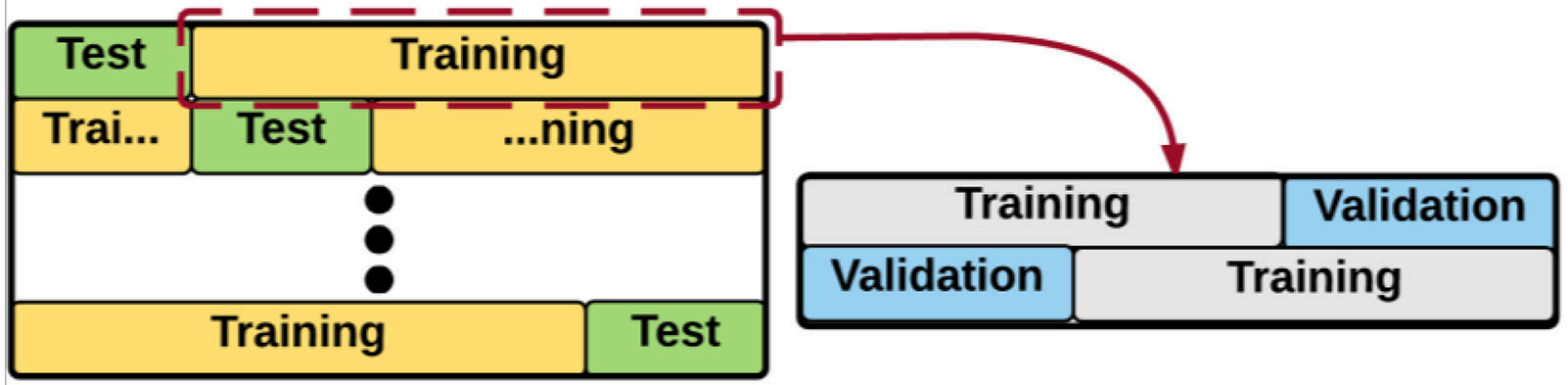
**Representation of the Nested Cross Validation (NCV)**. An outer cross validation module is used to evaluate the accuracy/error of an inner cross validation. In the inner cross validation module, the hyper-parameters are tuned or the features are selected.

We used nested cross validation for tuning the hyper-parameters of the model, selecting the features, and evaluating the model. In machine learning, hyper-parameter tuning refers to the problem of finding the best set of parameters for a model (Bergstra and Bengio, 2012). As seen in Figure 5, the module automatically performs an exhaustive search over various parameter values for an estimator and finds the best performing set of parameters for the given estimator, using the grid search approach (Chang and Lin, 2011). In this approach, a (multi-dimensional) grid of parameters is defined for the estimator and the cross validation technique is used for searching over the grid, and identifying the best performing set of parameters. After finding the best set of parameters for the estimator, we selected the best set of features automatically; using another five-fold nested cross validation module, and performed the classification tasks, i.e., AD-CT, AD-MCI, MCI-CT. In five-fold cross validation, the classification is performed in five iterations, separately for each pair-wise class combination of patients, i.e., AD-CT, AD-MCI, MCI-CT. Each iteration was divided into three steps: feature selection/hyper-parameter tuning, classifier training and classification. Feature selection/hyper-parameter tuning and classifier training was performed using (*5* − *1* = *4*) data folds per class, leaving out one-fold per class for subsequent classification. This process was done five times in order to apply classification to all the subjects. We implemented a five-fold nested cross validation (NCV) as the training phase included setting the hyper-parameters of the model and selecting the features. Through NCV (Figure 5), an outer cross validation was used to evaluate the accuracy and performance of an inner cross validation in which the hyper-parameters were tuned or the features were selected for the learning process. Thus, NCV helps to obtain an unbiased estimation of the true error and performance of the estimator (Varma and Simon, 2006).

#### Hyper-parameter setting

Hyper-parameter tuning is a crucial step in the model selection procedure. The task is to determine the best set of hyper-parameters for a given learning algorithm, through optimizing a performance measure, e.g., accuracy or error (Bergstra and Bengio, 2012). We used grid search method for hyper-parameter tuning. In the grid search method, a search is performed on a grid of parameters created based on a pre-defined subset of hyper-parameter space of a learner, in an aim to find the set of hyper-parameters that maximizes the performance of the learning algorithm. The grid search was implemented as part of a five-fold nested cross validation module, explained before, through which the accuracy of the learner was evaluated.

#### Feature selection

**Figure 6:**
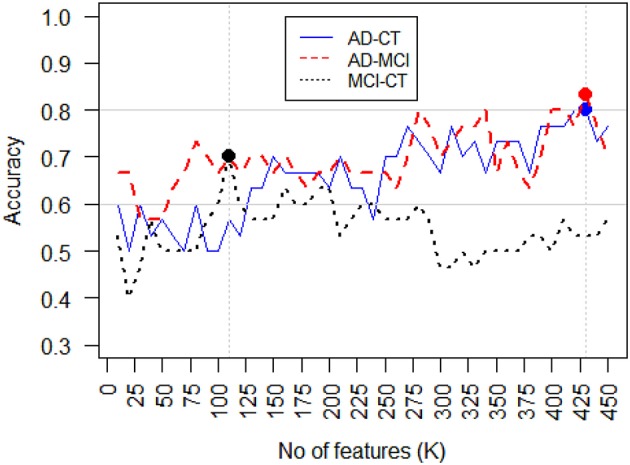
**Classification accuracy vs. feature selection threshold, i.e., the number of features selected (***K***)**. For AD-CT and AD-MCI classification task, the best *K* = 430, features maximize the accuracy of the classifier. The best *K*-value found for MCI-CT classification was *K* = 110.

Feature (attribute) selection is an approach to identify relevant features and reduce the noise in order to increase signal-to-noise ratio and reduce overfitting, by constructing a generalized model through the selections of a subset of features from the original feature set (Bermingham et al., 2015). The central assumption when using a feature selection technique is that the data contains many redundant or irrelevant features. Redundant features are those which are repeated and provide no extra information compared to the currently selected features, while irrelevant features are those which do not provide any useful information. Feature selection techniques are often used in domains where there are many features and comparatively few samples, as is our problem. We implemented a nested cross validation module to test various feature selection methods for all the three classification tasks, i.e., AD-CT, AD-MCI, and CT-MCI. Amongst all the tested feature selection approaches, the *K*-best features outperformed others. In *K*-best feature selection, the features are ranked based on their power in performing the classification, and then the top *K* features are selected for the given estimator. The features were ranked based on the ANOVA *F*-value between features. The number of top features, i.e., *K*, should be manually defined. We tested various *K*s and evaluated the classification accuracy in each of the three classification tasks, Figure 6, shows the classification accuracy vs. the best number of features to include in the classifier (*K*), for different classification tasks. As seen, *K* = 430 maximizes the classification accuracy in both AD-CT and AD-MCI classification tasks. And, *K* = 110 was found to be the best number of top features to include in the MCI-CT classifier. The features were selected based on their score and *p*-value. The selected top-*K* features for each of the classification tasks were considered in the respective classifier, which will be explained in detail in the next part.

#### Classification

**Figure 7:**
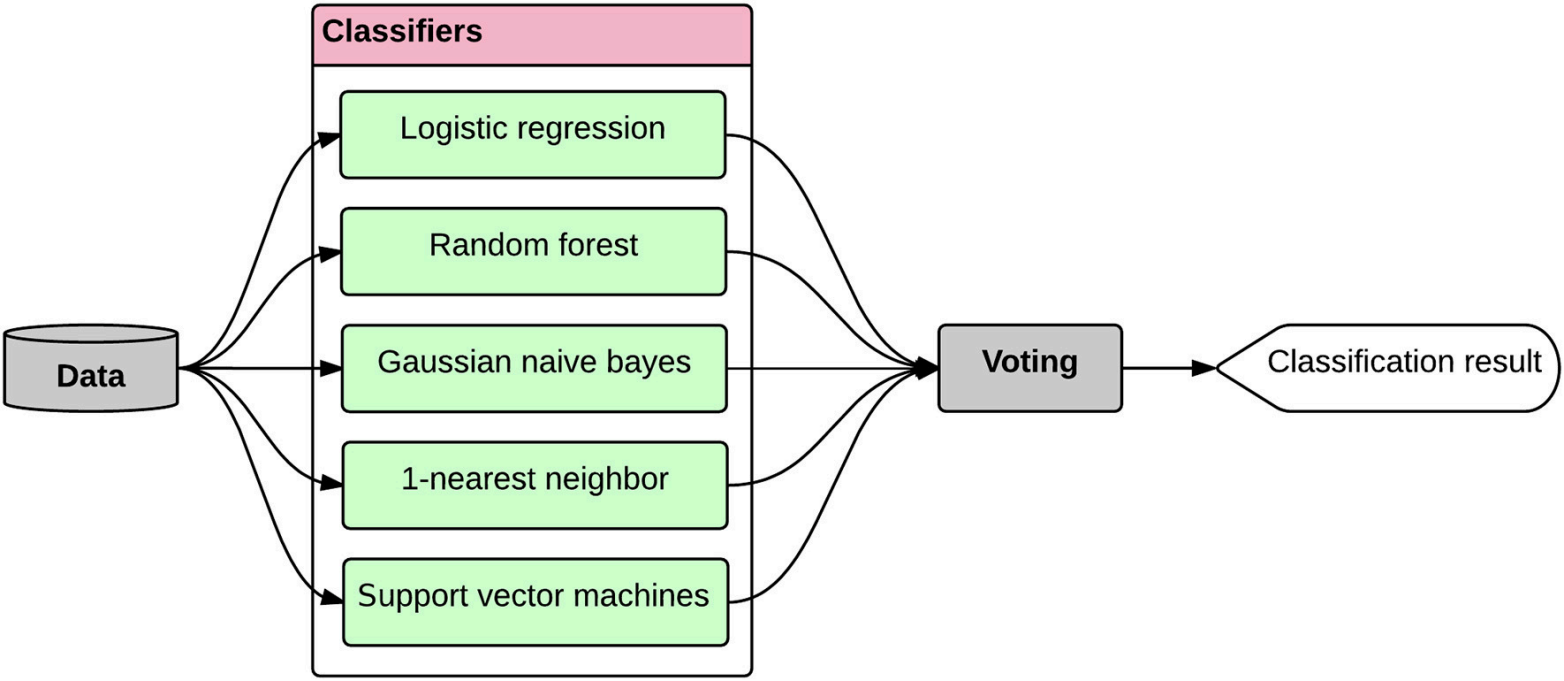
**The ensemble classifier design**. The data is provided to a set of diverse base classifiers and their decisions are then combined through a voting procedure to obtain the final classification result. The whole process was performed within a nested five-fold cross validation module.

As mentioned, we considered pair-wise class combinations of subjects and defined three separate classification tasks, i.e., AD-CT, AD-MCI, and CT-MCI. After tuning the hyper-parameters and selecting the most informative features, the classification was done using the ensemble classification method. In ensemble learning, multiple classifiers are used such that the ensemble classification system outperforms all of the constituent classifiers (Opitz and Maclin, 1999; Dietterich, 2000). Ensemble classifiers become even more advantageous if classifiers with different decision boundaries are used, allowing for more flexibility through promoting diversity among the models (Brown et al., 2005). The ensemble learning is very similar to human behavior in making important decisions. Especially in case of medical diagnosis, humans prefer to increase the reliability of their decision through asking the opinion of various doctors (Sesmero et al., 2015). We simulated the same process in our system by generating a diverse set of base classifiers whose decision boarders complement each other, and combining their outputs such that the accuracy of the classification was improved (Figure 7). Thus, the ensemble learner was built in two phases: (1) generating a set of diverse base classifiers, and (2) combining the decisions made by the base classifiers to obtain one decision.

The diversity of the base classifiers is of high importance. The diversity of two given classifiers is high if they result in errors at different samples (Sesmero et al., 2015). If a pair of classifiers is not diverse, then the decision made by each of them might be similar, therefore, the final ensemble decision will not improve. But if the decision boarders of a pair of classifiers complement each other, we can expect an improved performance for the ensemble classifier. Although the diversity of base learners is a mandatory condition for a good ensemble, there is no consensus on quantifying the diversity (Kuncheva and Whitaker, 2003). We considered pair-wise combinations of the base classifiers candidates and calculated the Q metric as a proxy for diversity. Equation (10) shows the definition of the Q metric (Sesmero et al., 2015).

(10) $$Q=\frac{N^{11}N^{00}-N^{01}N^{10}}{N^{11}N^{00}+N^{01}N^{10}}.$$

In Equation (10), *N*^*ij*^ is the number of samples that were classified correctly (*i* = 1) or incorrectly (*i* = 0) by the first classifier in the pair, and correctly (*j* = 1) or incorrectly (*j* = 0) by the second classier in the pair. The lower the Q is for a pair of base classifiers, the more diversity the pair has. We tested various combinations of base learners and found that the combination of logistic regression, random forest, gaussian naïve bayes, 1-nearest neighbor, and support vector machines performs the best for the defined classification tasks. We then used voting for combining the decisions made by the base classifiers and obtaining the final classification result (Figure 7). In voting approach, the final classification decision on a new sample, i.e., *C(x)*, is made through voting on all the base classifiers (*BC*_*i*_), each having a weight of *w*_*i*_, as stated in Equation (11) (Rokach, 2010).

(11) $$C(x)=sign\left(\underset{i\;=\;1}{\sum^{n}}w_{i}.\;BC_{i}\right).$$

In Equation (11), *n* is the number of base classifiers (in our case, *n* = 5). The preliminary weights of the base classifiers were first generated within the hyper-parameter setting procedure. We then refined the weights by testing various combinations of weights and determined the best weighting set for each of the classification tasks. Intuitively, we assigned larger weights to more accurate classifiers, or set of classifiers with complementary decision boarders. The entire classification process was performed within a nested five-fold cross validation module, as explained before in the cross validation section. The classification module generates a positive (+1) or negative (−1) label for each subject corresponding to the input labels, e.g., AD = +1 and CT = −1 in AD-CT task. After that, this output can be compared with the input label (neuropsychological diagnosis) in order to measure the success level of the approach. Success in classification is achieved when the output label from the classifier is equivalent to the input label. Accuracy of classification is the percentage of correctly predicted (or detected) output labels for all subjects from both positive and negative input classes.

## Results

We evaluated our model on 45 subjects, comprising 15 AD patients, 15 MCI patients, and 15 healthy volunteers. A total number of 451 features was calculated for each subject from which 430, 430, and 110 features were selected as the most informative features for AD-CT, AD-MCI, and MCI-CT classification tasks, respectively. Table 3 shows the performance metrics calculated for the base classifiers as well as the ensemble technique with and without feature selection module. The feature selection module was considered for all the base classifiers during the evaluation stage, as it improved their performance. As seen in Table 3, the ensemble framework with feature selection outperforms all the other listed classifiers, and shows a promising performance. The ensemble framework with feature selection resulted in a classification accuracy of 83.3% for AD vs. MCI, 80% for AD vs. CT, and 70% for MCI vs. CT. Accuracy of the classification was defined as the number of correct predictions divided by the total number of predictions. We also checked for the recall metric defined as the number of true-positives divided by the number of true-positives and false-negatives, i.e., *Recall* = *TP*/(*TP*+*FN*). According to Table 3, the recall is 80, 80, and 50% for AD-CT, AD-MCI, and MCI-CT classification tasks, respectively. Recall can be considered as a measure of completeness of a classifier such that a low recall can indicate the high number of false-negatives. Recall can be also regarded as a sensitivity measure since it evaluates the effectiveness of a classifier in identifying the positive labels (Sokolova and Lapalme, 2009). Finally, we checked the F-1 score of the classifiers. The F-1 score is useful if we want to select a classification model such that it holds a balance between precision and recall. Theoretically, the F-1 score is defined as the harmonic mean of precision and recall, i.e., 2(*Precision* ^*^ *Recall*)/(*Precision*+*Recall*), in which the precision is the number of true-positives divided by true-positives and false-positives. As it is seen in Table 3, the F-1 score of the ensemble framework with feature selection is 79.8, 82.5, and 66.7% for AD-CT, AD-MCI, and MCI-CT classification tasks.

**Table 3:** **Performance results of the base classifiers as well as the ensemble in AD-CT, AD-MCI, and MCI-CT classification tasks**.

**Base Classifiers**	**Classification Task**								
	**AD-CT**			**AD-MCI**			**MCI-CT**		
	**Acc**.	**Recall**	**F-1**	**Acc**.	**Recall**	**F-1**	**Acc**.	**Recall**	**F-1**
Logistic Regression	0.733	**0.867**	0.767	0.7	**0.8**	0.729	0.667	**0.667**	**0.667**
Gaussian Naïve Bayes	0.567	0.6	0.566	0.6	0.6	0.584	0.633	0.6	0.58
Support Vector Machines	0.767	0.8	0.779	0.7	0.733	0.72	0.533	0.533	0.518
Random Forests	0.43	0.2	0.26	0.633	0.467	0.491	0.567	0.533	0.542
1-Nearest Neighbor	0.633	0.533	0.58	0.667	0.467	0.531	0.6	0.6	0.578
Ensemble	0.733	0.733	0.731	0.767	0.733	0.758	0.5	0.467	0.48
Ensemble with Feature Selection	**0.8**	0.8	**0.798**	**0.833**	**0.8**	**0.825**	**0.7**	0.5	**0.667**

*All the listed classifiers were tested within a five-fold cross validation module*.

*Feature selection was used for all the base classifiers as it improved their performance*.

*The best results for each column were bolded*.

We further investigated the performance of the ensemble classifier with feature selection by calculating the confidence intervals of various performance metrics. We ran the cross validated ensemble classifier for 100 times for each performance metric, i.e., accuracy, recall (sensitivity), specificity, F-1 score, and receiver operating characteristic area under the curve (ROC AUC), and each of the classification tasks, i.e., AD-CT, AD-MCI, and MCI-CT, and stored the result. Next, we checked whether the performance metric is normally distributed. As expected, we observed that none of the metrics for the classification tasks are normally distributed. Therefore, we used the statistical median for calculating the confidence intervals^2^. Table 4 presents the results for 95% confidence intervals. It shows that we can be 95% confident that the true median of the population is in the range of [0.743, 0.757], [0.759, 0.774], and [0.662, 0.671] for AD-CT, AD-MCI, and MCI-CT classification tasks, respectively. Although the highest accuracy is still observed for AD-MCI classification task, it is very close to the confidence interval of AD-CT classification. Additionally, it was observed that the model is the best in detecting the positive events for AD-CT classification with recall of 0.8.

**Table 4:** **Accuracy metric 95% confidence intervals for the ensemble classifier with feature selection in AD-CT, AD-MCI, and MCI-CT classification tasks**.

		**Classification task**		
		**AD-CT**	**AD-MCI**	**MCI-CT**
Median [95% Confidence Interval]	Accuracy	0.75 [0.743, 0.757]	0.767 [0.759, 0.774]	0.667 [0.662, 0.671]
	Recall	0.8 [0.792, 0.808]	0.73 [0.718, 0.742]	0.6 [0.589, 0.611]
	Specificity	0.67 [0.617, 0.723]	0.67 [0.626, 0.714]	0.43 [0.384, 0.476]
	F-1	0.77 [0.763, 0.777]	0.74 [0.729, 0.751]	0.56 [0.553, 0.567]
	ROC AUC	0.76 [0.754, 0.767]	0.78 [0.775, 0.785]	0.56 [0.557, 0.563]

We checked for the top-5 features that were detected within the ensemble classification process for each of the classification tasks, separately. For AD-CT classification task, betweenness centrality at Brodmann Area 2 (primary somatosensory cortex), eigenvector centrality at Brodmann Area 1 (primary somatosensory cortex), load centrality at Brodmann Areas 2 (primary somatosensory cortex) and 27 (piriform cortex), and closeness centrality at Brodmann Area 1 (primary somatosensory cortex) were detected as the top-5 most important features. Katz centrality at Brodmann Area 3 (primary somatosensory cortex), degree and closeness centrality at Brodmann Area 5 (somatosensory association cortex), node redundancy coefficient and load centrality at Brodmann Area 4 (primary motor cortex) were found as the most important features for AD-MCI classification. For MCI-CT classification, we observed the hit centrality, page rank, betweenness centrality, and load centrality at Brodmann Area 6 (premotor cortex) along with hub centrality at Brodmann Area 1 (primary somatosensory cortex) to be the most important features.

## Discussion

Our results indicate that complex graph measures can be used as makers to diagnose Alzheimer's disease. Moreover, these results indicate that Alzheimer's disease affects Brodmann areas in the sensorimotor cortex and piriform cortex, where we found abnormalities in out measures. The piriform cortex, whose load centrality has been shown as a good discriminative feature for Alzheimer's disease diagnosis by our results, has an important role in olfactory perception (Howard et al., 2009). The piriform cortex is located at the junction of the temporal and frontal lobes and is the neighbor of the entorhinal cortex (Mai et al., 2008). The entorhinal cortex is known in the literature as one of the first affected areas in Alzheimer disease (Khan et al., 2013). Additionally, the primary motor cortex is significantly involved in late and terminal stages of Alzheimer's disease (Suva et al., 1999).

In our study, node redundancy coefficient and load centrality in the primary motor cortex were recognized as good indicators of Alzheimer's disease in contrast to MCI. In relation to these findings in the primary motor cortex (and in other brain regions), it is important to point out that abnormalities in node redundancy coefficient and load centrality do not mean variations in the connectivity at that exact location. Degree is the only network measure that is directly correlated to connectivity changes in the specific node (in this case primary motor cortex). As Degree is not one of the abnormal measures, it indicates that abnormal connectivity patterns at primary motor cortex are not a relevant feature for AD diagnosis. The relevant network measures revealed here (node redundancy coefficient and load centrality) indicate that connectivity abnormalities on AD are occurring in parallel (in circuit terms) in brain pathways that cross primary motor cortex, which together generate these network patterns found at the primary motor cortex. In other words, the primary motor cortex does not represent the primary effect, rather than the secondary effect of the pathology.

Our findings identified measures in the primary sensory cortex (betweenness centrality, eigenvector centrality, load centrality, closeness centrality, and Katz centrality), somatosensory association cortex (degree and closeness centralities) and primary motor cortex (node redundancy coefficient and load centrality) as good discriminative features for Alzheimer's disease diagnosis, while measures at the premotor cortex (HITS centrality, page rank, betweenness centrality, and load centrality) were identified as good discriminative features for MCI diagnosis^3^.

Therefore, load centrality, betweenness centrality and closeness centrality were found to be the most relevant network measures in our study, as they were the top identified features at different nodes. Other measures, as eigenvector centrality, Katz centrality, degree centrality, node redundancy coefficient, HITS centrality, page rank and hub centrality were also recognized as relevant features for classifications among Alzheimer's disease patients, MCI patients, and healthy controls.

The accuracy value achieved in our study can be compared to what other researches performed using other modalities of MRI, such as structural (Wolz et al., 2011; Casanova et al., 2012; Lillemark et al., 2014), functional (Chen et al., 2011) and voxel-based diffusion (Dyrba et al., 2013). All those studies, as well as ours, achieved accuracies in the range of 80–90%. While structural MRI can reveal localized deformations, diffusion MRI based network analysis has the potential to help the development of new pathologic brain segmentation atlases grouping brain matter according to their network patterns. In this way, our paper shows that the use of graph measures as feature of diffusion MRI data can run in parallel to the application of other modalities toward finding biomarkers of AD. The combination of features from these different modalities may considerably increase the potential of the AD diagnosis. Therefore, the development of methods which efficiently combines these multimodal features is a field to be explored by next studies. Nevertheless, our results indicate that complex graph measures may effectively be used to diagnose Alzheimer's disease.

A limitation of this study is the small sample size (15 subjects for each of the three classes). Therefore, this study is a “proof of concept” about a procedure for the classification of MRI markers between AD dementia, MCI, and normal old individuals. The reliability of these results can be tested later in a complementary study based on a larger sample size (at least 100 subjects per class). It is also important to remark that the small sample of amnesic MCI could be a mix of people with AD and other dementing disorders, such as dementia with Lewy body dementia. Due to these facts, the moderate classification between control subjects and MCI in this study does not mean an ability of the procedure to disentangle between normality and the prodromal stages of AD. However, although the sample size is small, it is quite encouraging that the proposed ensemble classification framework which included a well-tuned feature selection component and was validated within a five-fold cross validation module, resulted in quite good classification performance. We expect larger sample would help the system to better distinguish between the AD and non-AD groups. A longitudinal study can assign at what time-point in the evolution of the disease each region is more likely to be affected by Alzheimer's disease. Moreover, longitudinal studies may have the potential to reveal first evidences of prodromal Alzheimer's disease. Finally, supervised classification approach requires a set of labeled observations (Bishop, 2007), thus making it highly dependent on the ground truth provided by the diagnosis of the clinicians. Assuming that there is a possibility of misdiagnosis, unsupervised classification techniques, such as K-Means clustering (Lloyd, 1982; Jain et al., 1999), can be used to improve the reliability of classifications.

## Ethics statement

This study was carried out in accordance with the recommendations of ‘International Guidelines for Biomedical Research involving human beings, Declaration of Helsinki’ and ‘Resolution 196/96, Brazilian National Health Council’. Being a retrospective survey of clinical files, patients did not sign an informed consent, in accordance to Brazilian regulations. The protocol was approved by the ‘Ethics Committee of D’Or Institute for Research and Education'.

## Author contributions

Conceiving and designing the experiments: AE, JD, RS, PR. Performing algorithmic experiments: AE, JD, DN. Analyzing the data: AE. Data/materials: JD, FT, IB, GC. Writing of the manuscript: AE, JD, DN, RS, PR.

### Conflict of interest statement

The authors declare that the research was conducted in the absence of any commercial or financial relationships that could be construed as a potential conflict of interest.

## Footnotes

^1^We used 1,000,000 seeds as starting fiber points for each individual brain. This produces a potential maximum of 1,000,000 deterministic fibers per brain (in practice we have much less due to minimum length constraint threshold). Using 1,000,000 fibers can balance the deterministic tractography for all the participants.

^2^To calculate the confidence interval for a mean, we either need a large sample size or a normally distributed population (Dean and Dixon, 1951; Moore, 2007).

^3^Please see Table A1 for statistical comparison of the key DTI biomarkers at the group level.

## Appendix

We statistically compared the key DTI biomarkers at the group level, i.e., AD vs. MCI, AD-CT, and MCI-CT. In particular, we performed the two-sample *t*-test (Snedecor and Cochran, 1989), for each of the key DTI biomarkers (e.g., primary somatosensory cortex-betweenness centrality) in each group (e.g., AD-CT group), to determine if the mean of the examined biomarker is equal for both samples (e.g., AD vs. CT). The results are shown in Table A1.

**Table A1:** **Statistical comparison of the key DTI biomarkers at the group level**.

	**Classification task**					
	**AD-CT**		**AD-MCI**		**MCI-CT**	
	**AD-CT Key Biomarker**	***t*****-statistic**	**AD-MCI Key Biomarkers**	***t*****-statistic**	**MCI-CT Key Biomarkers**	***t*****-statistic**
Null Hypothesis: There is no difference between the average of the key DTI biomarker in both samples.	Primary somatosensory cortex -betweeness centrality	−2.41^*^	Primary somatosensory cortex -katz centrality	−1.59	Premotor cortex-HITS centrality	−3.6^**^
	Primary somatosensory cortex-eigenvector centrality	−2.37^*^	Somatosensory association cortex-degree centrality	−1.54	premotor cortex -page rank	−3.44^**^
	Primary auditory cortex-load centrality	−2.36^*^	Somatosensory association cortex-closeness centrality	−1.51	Premotor cortex -betweenness centrality	−2.55^*^
	Primary somatosensory cortex -load centrality	2.26^*^	Primary motor cortex-node redundancy	−1.49	Premotor cortex -load centrality	−2.54^*^
	Primary somatosensory cortex -closeness centrality	−2.22^*^	Primary motor cortex-load centrality	1.42	Primary somatosensory cortex-HITS centrality	−2.32^*^

* *p < 0.05*,

** *p < 0.01*.

AD diagnoses were performed considering NINCDS-ADRDA criteria (Knopman et al., 2001). All individuals underwent a comprehensive evaluation for diagnostic propose, which included Mini Mental State Evaluation (MMSE), Span (digit and spatial) forward and backward, Clock Drawing Test (CDT), Verbal Fluency (semantic and letter), Family Pictures, Geriatric Depression Scale (Yesavage et al., 1983), Memory Assessment Complaints Questionnaire (Crook et al., 1992). The quantitative results for these tests are in the Table A2.

**Table A2:** **Quantitative results for diagnostic propose of AD and amnesic MCI**.

**Patient**	**CDT**	**Span forward**	**Span backward**	**Fluency**	**MMSE**	**Crook**	**Yesavage**	**Family pictures**	**Diagnosis**
Sub 01	10	4	4	16	28	30	6	30	Normal
Sub 02	10	5	4	12	26	21	2	29	Normal
Sub 03	10	6	4	14	26	32	5	26	Normal
Sub 04	10	6	4	21	25	35	10	26	Normal
Sub 05	10	5	3	8	22	29	1	24	Normal
Sub 06	10	4	2	11	21	31	4	20	Normal
Sub 07	10	5	4	24	29	27	0	30	Normal
Sub 08	10	6	3	12	29	21	4	29	Normal
Sub 09	10	3	3	19	29	31	8	27	Normal
Sub 10	10	4	4	12	26	29	11	30	Normal
Sub 11	8	7	5	19	25	29	7	25	Normal
Sub 12	10	5	3	11	29	26	4	23	Normal
Sub 13	10	4	4	16	28	33	3	27	Normal
Sub 14	10	6	5	21	29	27	0	27	Normal
Sub 15	9	4	3	11	25	23	5	24	Normal
Sub 16	10	5	4	15	26	31	6	28	Amnestic MCI
Sub 17	10	5	4	13	27	35	12	30	Amnestic MCI
Sub 18	10	5	4	10	27	31	13	30	Amnestic MCI
Sub 19	10	4	3	13	30	26	4	22	Amnestic MCI
Sub 20	8	4	4	6	20	30	2	27	Amnestic MCI
Sub 21	4	6	4	9	25	27	3	18	Amnestic MCI
Sub 22	10	5	4	19	27	28	1	30	Amnestic MCI
Sub 23	10	5	3	13	24	34	6	22	Amnestic MCI
Sub 24	10	4	2	17	27	23	4	24	Amnestic MCI
Sub 25	10	6	3	18	29	31	3	26	Amnestic MCI
Sub 26	10	4	2	10	24	25	3	23	Amnestic MCI
Sub 27	7	4	4	16	25	24	8	17	Amnestic MCI
Sub 28	2	5	3	13	28	26	11	17	Amnestic MCI
Sub 29	10	6	4	10	26	21	11	18	Amnestic MCI
Sub 30	10	4	3	13	23	27	12	15	Amnestic MCI
Sub 31	9	4	3	9	17	24	4	16	AD dementia
Sub 32	7	4	4	10	21	35	8	13	AD dementia
Sub 33	3	3	0	6	14	28	0	13	AD dementia
Sub 34	5	4	3	9	25	26	2	23	AD dementia
Sub 35	10	7	4	15	25	30	4	15	AD dementia
Sub 36	10	5	4	10	26	30	3	19	AD dementia
Sub 37	10	5	3	9	24	22	0	21	AD dementia
Sub 38	3	5	3	5	23	25	1	15	AD dementia
Sub 39	2	5	2	5	15	9	2	16	AD dementia
Sub 40	10	4	2	11	22	32	7	15	AD dementia
Sub 41	4	4	3	9	21	21	0	19	AD dementia
Sub 42	10	3	3	7	23	23	3	13	AD dementia
Sub 43	10	5	3	14	28	35	7	21	AD dementia
Sub 44	3	4	2	0	12	23	2	16	AD dementia
Sub 45	9	4	3	12	25	34	4	20	AD dementia
